# Effects of Must Settling and Pre-Bottling Filtration in Ancestral Sparkling Wine Production

**DOI:** 10.3390/foods15152661

**Published:** 2026-07-29

**Authors:** Arnau Just-Borràs, Nadia Gregori, Antoni Canalda, Jordi Gombau, Pedro Cabanillas, Richard Marchal, Cristina Ubeda, Joan M. Canals, Fernando Zamora

**Affiliations:** 1 Departament de Bioquímica i Biotecnologia, Facultat d’Enologia de Tarragona, Universitat Rovira i Virgili, C/Marcel.li Domingo 1, 43007 Tarragona, Spain; arnau.just@fundacio.urv.cat (A.J.-B.); nadia.gregori@urv.cat (N.G.); antoni.canalda@urv.cat (A.C.); jordi.gombau@urv.cat (J.G.); pedro.cabanillasas@urv.cat (P.C.); jmcanals@urv.cat (J.M.C.); 2 Laboratoire Innovation Vigne et Œnologie (LIVO), Faculté des Sciences, Université de Reims Champagne-Ardenne, B.P. 1039, 51687 Reims Cedex 02, France; richard.marchal@univ-reims.fr; 3 Departamento de Nutrición, Bromatología, Toxicología y Medicina Legal, Facultad de Farmacia, Universidad de Sevilla, C/P. García González No 2, 41012 Seville, Spain; c_ubeda@us.es

**Keywords:** ancestral method, sparkling wine, settling, filtration, foaming properties, volatile profile

## Abstract

The ancestral method of sparkling wine production requires no sugar addition and is based on a single alcoholic fermentation that finishes in the bottle. It has recently gained popularity among both natural and conventional producers due to its capacity to expand sparkling wine portfolios and its advantages for sparkling wine production in warm regions such as the Mediterranean basin. However, some technological and other key aspects of the process remain poorly defined. This study investigates how must settling (using pectolytic enzymes) and pre-bottling filtration influence the physicochemical composition, colloidal structure, foaming properties, volatile profile, and sensory attributes of ancestral sparkling wines. Macabeo grapes (2023 vintage) were vinified under four treatment combinations of these two operations, and the wines were analysed after eighteen months of lees ageing. Significant differences were observed in total polyphenol index, titratable acidity, macromolecular composition, and colloidal particle concentration. Non-settled wines exhibited higher colloidal loads and lower foamability, whereas settling and filtration improved foam height and stability. Volatile (GC-Headspace) analysis showed that settled wines contained higher concentrations of fruity and floral esters, while non-settled and non-filtered wines had greater levels of volatile phenols and volatile acids associated with unpleasant aromas. Sensory assessment confirmed perceptible differences between treatments, particularly in phenolic, fruity/floral, and Maillard reaction aromaticity. These findings provide insights into the impact of two critical oenological practices on wine stability and aroma and provide technologically relevant guidance for optimizing quality in this emerging sparkling-wine category.

## 1. Introduction

There is increasing interest in producing wines using alternative oenological approaches, such as biodynamic, organic farming or natural wine production [1]. Although some of these practices are legally regulated, others (such as *natural wine*) are vague and ambiguously defined in most countries [2]. Winemaking practices typically associated with this natural wine movement include the avoidance of settling, filtration, and sulphur dioxide usage [3]. In the context of sparkling wines, the ancestral method has emerged as an alternative approach for diversifying product styles [4,5].

The ancestral method of sparkling wine production is based on a single fermentation that enables wineries to produce sparkling wines without the need of sugar addition [6]. Once fermentation has started, it is monitored until the concentration of residual fermentable sugars reaches approximately 16–18 g/L (roughly 1.0% (*v*/*v*) of ethanol) in order to obtain around 6 bar of CO_2_ pressure [7]. At this stage, the wine is bottled, primary fermentation is completed, and carbon dioxide overpressure is developed [8].

Although consumers may associate this method with natural wines, it is, in fact, a traditional winemaking practice that has long been employed by some conventional wine producers. Moreover, its use has recently expanded among many wineries, as it enables them to diversify and broaden their product portfolios [9]. Both conventional and natural wine producers face challenges stemming from the absence of a clear definition of the ancestral method, whose perceived “novelty” reflects limited knowledge of the process. This lack of background leads to uncertainty for wineries over which oenological practices may enhance the quality of their products [8].

This study focuses on two main oenological practices in this context: must settling and pre-bottling filtration. Must settling is common in conventional winemaking. With this process, winemakers avoid the presence of solid grape-derived particles in suspension during fermentation [10]. By eliminating these particles, undesired aromatic compounds and pesticides are avoided and the stability and quality of the product are protected [11]. However, excessive clarification during settling may lead to stuck and sluggish fermentations due to a lack of nutrients in the must [12].

In a previous study we analysed the role of pre-bottling filtration with the ancestral method [7]. This partial filtration step reduces the yeast population inoculated inside the bottle, thereby slowing down the fermentation kinetics. The lower yeast population also facilitates disgorging and reduces the risk of defects associated with coarse and excessive lees [13]. This step is not required with other methods of sparkling wine production such as the traditional method since a stabilization step is carried out between the first and second fermentations [12].

Given the limited information available and the growing interest among wineries in implementing best oenological practices for high-quality sparkling wine production using this recovered method, the present study compares the physicochemical composition and sensory attributes of ancestral sparkling wines that have or have not undergone must settling prior to fermentation and have or have not been filtered before bottling.

## 2. Materials and Methods

**Figure 1:**
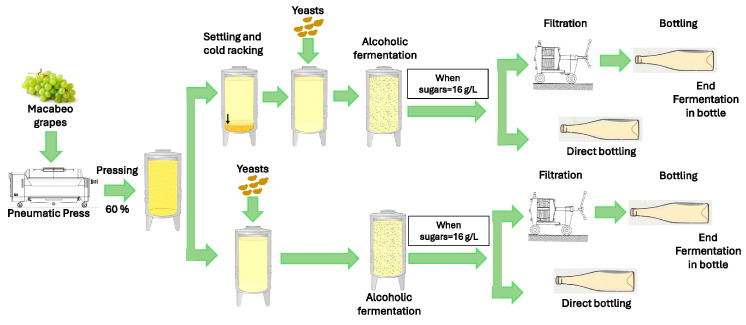
Experimental design.

This experiment was conducted using Macabeo grapes during the 2023 vintage at the experimental winery of Universitat Rovira i Virgili (Mas dels Frares, Constantí, AOC Tarragona, Spain). The grapes were harvested manually on 31 August, at a maturity stage corresponding to 10.4% potential alcohol, pH 3.00, and a titratable acidity of 3.7 g/L (expressed as tartaric acid). Figure 1 illustrates the experimental design. The grape bunches (650 Kg) were directly pressed in a pneumatic press (M5, Marzola, Navarrete, Spain) until a yield of 0.6 L/kg was obtained. The must was acidified with 1.5 g/L of L-tartaric acid due to its low titratable acidity and supplemented with 200 mg/L of yeast nutrients (Nutrient Vit, Lallemand Inc., Montreal, QC, Canada).

The must was divided immediately into two batches of 195 L each. One batch was supplemented with 20 mg/L of pectolytic enzyme (C-max, Lallemand Inc., Montreal, QC, Canada) to hydrolysate any pectic substances present, and with 50 mg/L of sulphur dioxide to facilitate settling. The must was then cold settled (8 °C) for 24 h. After settling, 170 L of clarified must were racked into a stainless-steel tank and immediately inoculated with 200 mg/L of a commercial strain of *Saccharomyces cerevisiae* (Lalvin EC1118™, Lallemand Inc., Montreal, QC, Canada). The temperature was maintained at 16–18 °C, and the fermentation kinetics were monitored using a digital densimeter (Mettler Toledo-PortableLab™, Cornellà de Llobregat, Barcelona, Spain). When the must densities approached 1005 kg/m^3^, analytical monitoring was initiated to determine the precise time at which residual fermentable sugars reached the appropriate level for bottling (16.0 g/L). Once the fermenting must reached that value, it was racked and cooled to 5 °C to slow down alcoholic fermentation. The fermenting must was then divided into two parts of roughly 85 L. One of these parts was directly bottled to complete the first fermentation inside the bottle. The other part was filtered with a 310 mm diameter plate filter (Cristalinox 310 mm, In Via, Sant Sadurní d’Anoia, Barcelona, Spain) using paper filter sheets (FIBRAFIX^®^ AF 70, Filtrox, Santa Perpètua de Mogoda, Barcelona, Spain) with a nominal pore size of 1.5–3.0 µm to reduce the yeast population.

The second batch of grape must obtained after pressing was immediately inoculated with 200 mg/L of the same commercial *Saccharomyces cerevisiae* strain without prior settling and, consequently, without the addition of sulphur dioxide or pectolytic enzymes to reproduce the conditions employed in some wineries. The absence of these additives may have had a significant influence on the final composition and sensory attributes of the resulting wines. The same procedure as for the first experimental group was then followed. Briefly, when the must densities approached 1005 kg/m^3^, monitoring was performed to determine the appropriate bottling point. The must was then racked, cooled, and divided into two parts, one of which was bottled to complete the fermentation in the bottle, and the other was filtered to reduce the yeast population prior to bottling.

The four experimental samples were supplemented with 30 mg/L of adjuvant 92 (Station Oenotechnique de Champagne, Epernay, France) prior to bottling to facilitate the riddling process. All the fermenting musts were then bottled, crown sealed and stored at 15–16 °C until disgorgement. The yeast populations at bottling were as follows: 15–20 × 10^6^ cells/mL for the filtered groups and 30–40 × 10^6^ cells/mL for the non-filtered groups.

Fermentation kinetics were monitored by measuring the accumulated pressure inside the bottle using a non-invasive method (L. sensor CO_2_, FT System, Alseno, Italy). All sparkling wines exhibited regular fermentation kinetics, reaching completion after approximately 30 days.

After eighteen months of ageing at 16 °C, six bottles from each experimental group (24 bottles in total) were transferred to a pupitre, and the riddling process was performed manually. Once all the lees sediment had fully collected in the neck of the bottle (roughly 12 days), disgorging was performed manually after freezing their necks at −28 °C using a Champagel apparatus (Maquinària Moderna, Sant Sadurní d’Anoia, Barcelona, Spain). For each condition, physicochemical analyses were performed on three bottles and sensory evaluation on the other three. Reported values represent the mean of the three replicate bottles per condition.

A limitation of this study is that each winemaking treatment was carried out as a single fermentation without independent biological replicates of the initial vinification process. Replicating the complete experimental design, including the settling or non-settling of the must and the filtration or non-filtration of the wines, would have required a considerably more complex winemaking setup. Therefore, replication was performed using independent bottles of the finished wines for the analytical determinations. Although this approach allowed the treatments to be evaluated under controlled conditions, the lack of independent fermentation replicates limits the statistical strength of the results. Future studies should include biological replication of the complete vinification process to confirm these findings.

### 2.1. General Wine Parameters

The CO_2_ pressure inside the bottles was measured using a non-invasive Laser Sensor (L. sensor CO_2_, FT System, Alseno, Italy). For every other measurement, all wine samples were centrifuged at 13,000× *g* (Biofuge Primo centrifuge, Heraeus, Hanau, Germany) for 15 min at 4 °C to obtain clear samples and remove carbon dioxide. The ethanol content was determined by ebulliometry (GAB Analysis Systems, Moja-Olerdola, Barcelona, Spain). The concentrations of residual fermentable sugars (D-glucose and D-fructose), ammonium, primary amino nitrogen, glycerol, L-(+)-tartaric, L-malic, L-lactic, D-lactic, citric, gluconic and acetic acids were measured using commercial enzymatic kits (Biosystems, Barcelona, Spain) [14]. Titratable acidity and pH were determined following OIV-recommended methods [15]. Total sulphur dioxide content was determined using a commercial kit (GAB Analysis Systems, Moja-Olerdola, Barcelona, Spain).

### 2.2.  Proteins and Polysaccharides Quantification by HPLC

Protein measurement was processed and analysed by HRSEC-DAD using the methodology described by [16,17]. Polysaccharide measurement was processed and analysed by HRSEC-RID using the methodology described by [18].

### 2.3.  Volatile Compounds Extraction and Analysis by HS/SPME/GC/MS

The basic conditions of sample extraction were based on the following validated method previously reported by [19]: 7.5 mL of wine, 1.5 g of NaCl and 10 μL of 4-methyl-2-pentanol as an internal standard (IS) (0.75 mg/L) were placed into a 20 mL glass vial positioned in the thermostatised autosampler tray at 20 °C for HS-SPME sampling. GC–MS analysis was performed using an 8890 Agilent GC system coupled with an Agilent 5977B quadrupole mass spectrometer (Agilent, Santa Clara, CA, USA). A J&W CPWax-57CB capillary column (50 m × 0.25 mm) with a film thickness of 0.25 μm was used (Agilent, Santa Clara, CA, USA), with helium carrier gas at a flow rate of 1 mL/min. The temperature ramp employed for the blank runs was: 35 °C for 1 min, raised to 220 °C at 10 °C/min and held for 5 min. The oven temperature programme was as follows: 35 °C for 1 min, raised to 160 °C at 2.5 °C/min (held for 1 min), and raised again to 220 °C at 5 °C/min, with a final GC time of 64 min. The electron ionisation mass spectra (29 to 300 amu) were acquired in full scan mode at 70 eV. The samples were analysed twice (analytical replicates) and in triplicate (biological replicates), and blank runs with empty glass vials were performed after each analysis. All data were recorded using MS ChemStation software version LTS 01.11 (Agilent, Santa Clara, CA, USA).

Identification was carried out calculating linear retention indices (LRIs) injecting a solution of C_10_–C_40_ alkanes with the same conditions as sample analysis. The identification was made by matching the LRIs of each compound from the standard NIST library (2.0 version) found in the literature (Pherobase: www.pherobase.com (accessed on 25 January 2026).; NIST Mass Spectrometry Data Center). Data shown in this work were expressed as semiquantitative data expressed as the relative area with respect to 4-methyl-2-pentanol (IS).

### 2.4. Foaming Properties

A Mosalux device (Station Oenotechnique de Champagne, Epernay, France) was used to measure HM, the max height of the foam after CO_2_ injection through the glass frit, and HS, the stable height during CO_2_ injection. HM represents foamability (the wine’s ability to foam) while HS represents foam stability (the persistence of the foam collar or the wine’s ability to produce a stable foam). HM and HS are expressed in millimeters.

Sparkling wine samples were degassed by centrifugation (17,000× *g* for 15 min) and tempered at 18 °C for 24 h before analysis. The foam properties were measured using the Mosalux method [20,21]. A glass cylinder placed on a glass frit was filled with 100 mL of the sample. CO_2_ was then injected into the glass cylinder through the glass frit with a constant gas flow of 115 mL/min under a constant pressure of 2 bar.

### 2.5. Colloidal Parameters

Nanoparticle tracking analysis was performed to determine the concentration and size of the colloidal bodies of the samples using NanoSight Pro (Malvern, Worcestershire, UK), following a variation in the procedure described by [22].

Briefly, wine samples were degassed by centrifugation (17,000× *g* for 15 min) and then directly injected into the sample chamber using a sterile 1 mL syringe. Analysis was performed under a constant and controlled sample flow at room temperature. For each sample, five independent 60 s video replicates were recorded. Data acquisition and processing were automated to eliminate user subjectivity via NS XPLORER software v1.0.8641.14.

### 2.6.  Sensory Analysis

All sparkling wines were tasted by a 15-person trained panel comprising nine males and six females aged between 22 and 60. Tasting was conducted in the tasting room of the Faculty of Oenology of Tarragona (Universitat Rovira i Virgili), which was designed in accordance with UNE 87004.197 [23]. This sensory evaluation procedure was divided into three sessions to avoid sensorial fatigue.

The first two sessions comprised a series of triangular tests carried out using ISO official tasting glasses [24]. The volume served was approximately 20 mL and the service temperature was 6–8 °C. All six possible combinations were tested.

A third session for descriptive analysis was held. The tasters evaluated the intensity of seven sensory attributes (bubble size, bubble stability, phenolic aroma–animal nuances, fruity/floral aroma, Maillard reaction product (MRP) aroma, gas aggressivity and acidity) on a scale of 0 to 10 (0 = small or absence; 10 = large or highly present).

One of the wines (settled + filtered) was designated as the control, and all data were normalised with respect to this reference following a baseline correction procedure to directly express how each attribute deviated from the control group. For each assessor *a*, wine *p*, and attribute *k*, normalized scores were computed as (∆*_a_*_,_*_p_*_,_*_k_* *=* y *_a_*_,_*_p_*_,_*_k_* − y *_a_*_,_*_C_*_,_*_k_*).

A sensory training session was held prior to evaluation to ensure that the panellists reached consensus on the criteria for each sensory attribute. Samples were presented in random order to minimise the influence of the tasting order.

### 2.7.  Statistical Analysis

The data shown are the arithmetic means of triplicates with the standard deviation for each parameter. One-way ANOVA and two-way ANOVA, Tukey comparison tests, and principal component analysis (PCA) were performed using XLSTAT Basic (Addinsoft, Paris, France). The results of the sensory assessment were analysed using PanelCheck V1.4.2 software (Nofima Mat, Technical University of Denmark and University of Copenhagen). Triangular test *p* values have been calculated using Triangle test calculator developed by Jangevaare (https://jangevaare.shinyapps.io/triangle/) (accessed on 25 February 2026). Differences were considered statistically significant when *p* < 0.05.

## 3. Results and Discussion

### 3.1.  General Wine Parameters

Table 1 shows the analytical results for the main chemical components. As all wines were produced from the same batch of grapes, no differences were observed in ethanol content, pH or gluconic acid concentration.

Total Polyphenol Index (TPI) was higher in the non-filtered groups. This effect is probably attributable to filtration, as certain polyphenols may be retained by the filters. Must settling had no effect on this parameter, possibly because the use of a riddling agent during tirage causes the partial absorption of polyphenols in suspension during remuage [25].

As expected, sulphur dioxide concentrations were higher in wines produced from settled grape juice, since 50 mg/L of SO_2_ was added during the settling step. In contrast, the other grape juice tank received neither sulphur dioxide nor clarification treatment.

Wines made without juice settling had lower residual sugars than settled wines, though all levels were very low. These results may be due to the probable higher nutrient content of non-settled grape juice, which promotes greater sugar consumption by yeasts [26]. Despite these results, all wines completed fermentation (≤1.0 g/L sugar), and the small differences are unlikely to have had a sensorial effect [27].

Although wines produced from non-settled must exhibited higher concentrations of L-malic and citric acids, their total titratable acidity (TTA) was lower than that of wines produced from settled must. This difference is mainly attributable to their lower tartaric acid concentration, which has a greater impact on TTA than the higher concentrations of the other two acids. These effects can be explained by the presence of grape solids in the bottle. Grape solids of pulp origin may release citric and L-malic acid into the wine. These solid particles may also release potassium, which could react with tartaric acid to promote potassium bitartrate crystallisation [12,28,29].

No L-lactic acid was detected in any sparkling wine, which indicates that no malolactic fermentation occurred inside the bottle, a notable result given the low sulphur dioxide levels in all experimental groups.

No differences were observed in glycerol, D-lactic acid, or acetic acid, which suggests that neither grape solids nor high yeast populations induce lactic or acetic taints.

Table 1 also shows the ammonia and primary amino nitrogen (PAN) concentrations. Ammonia concentrations were similar for all sparkling wines (roughly 27 mg/L) in all experimental groups, consistent with values typically observed in wines aged on lees [30].

The concentration of PAN, which comprises free amino acids and small peptides, was higher in both non-filtered groups, whereas the settled + filtered group exhibited lower PAN, probably due to the lower yeast population in the bottle. After filtration, the smaller yeast population must metabolise the same amount of sugars as in the other experimental conditions and therefore requires proportionally more nitrogen for growth and fermentation. In contrast, the non-filtered groups, which have higher yeast populations, display higher PAN concentrations, reflecting the greater availability of nitrogen for a larger number of cells [31].

Unexpectedly, must settling had no effect on residual PAN or ammonium in the final wines.

### 3.2. Colour Determination

When the CieL*a*b* colour space coordinates were calculated (Supplementary Table S1) for all samples, only small differences were observed.

### 3.3.  Quantification of Protein and Polysaccharide Concentrations

**Figure 2:**
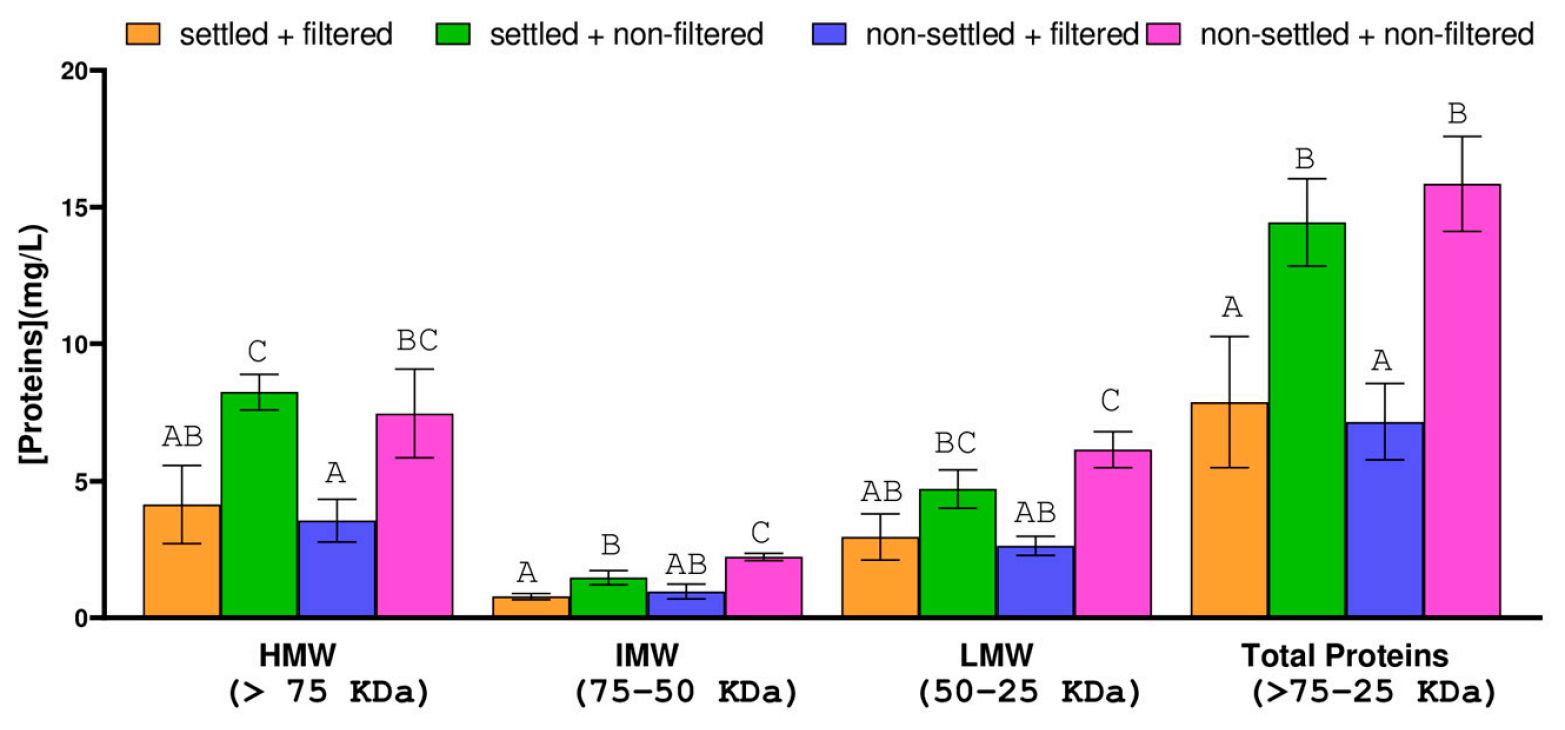
Protein composition. Results are expressed as mean ± SD of three replicates. Concentration of proteins is expressed as bovine serum albumin equivalents. HMW: High-molecular-weight Fraction; IMW: Intermediate-molecular-weight Fraction; LMW: Low-molecular-weight Fraction. Different letters indicate the existence of a statistical difference (*p* < 0.05).

Figure 2 presents the protein concentrations. All fractions exhibited similar trends throughout the study. In general, wines filtered prior to bottling showed lower total protein concentrations than unfiltered wines. This observation may be explained by differences in the yeast population present at the time of bottling, since a higher yeast cell concentration inside the bottle can lead to a greater release of intracellular compounds, including proteins, during the autolysis process [32]. Yeast autolysis is known to contribute significantly to the protein composition of sparkling wines during ageing on lees, thereby increasing the overall protein content of the final product.

In contrast, no significant differences attributable to must settling treatments were detected among the analysed wines. This lack of effect may be associated with the use of the adjuvant added at bottling, which could have minimized or masked potential differences derived from the settling process. Consequently, the influence of the initial clarification treatment on protein concentration appears to be less relevant than the contribution associated with yeast-derived compounds released during bottle ageing.

**Figure 3:**
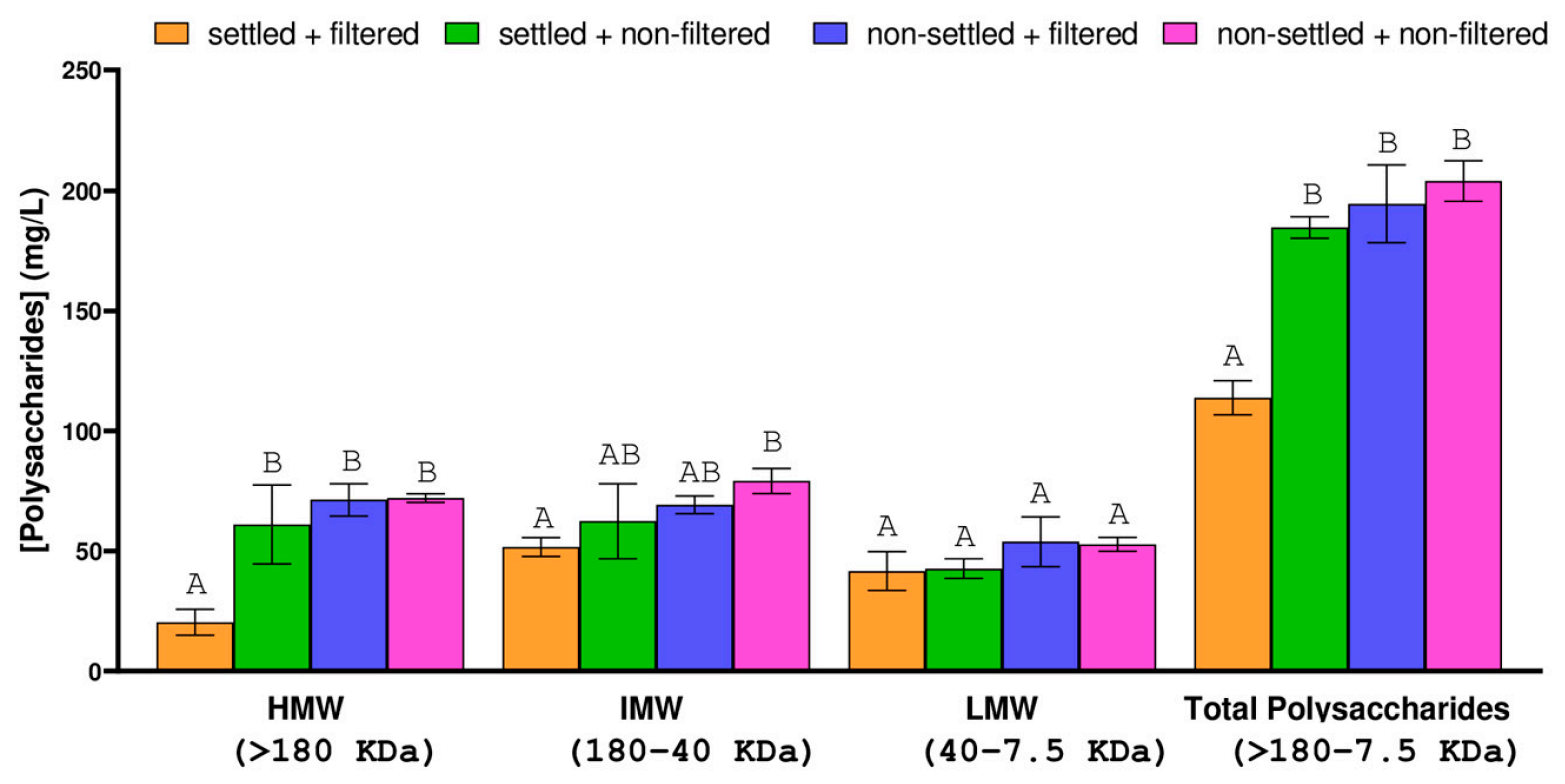
Polysaccharide composition. Results are expressed as mean ± SD of three replicates. Concentration of polysaccharides is expressed as pectin and dextran equivalents. HMW: High-molecular-weight Fraction; IMW: Intermediate-molecular-weight Fraction; LMW: Low-molecular-weight Fraction. Different letters indicate the existence of a statistical difference (*p* < 0.05).

Figure 3 shows the polysaccharide concentrations. Total polysaccharide levels were lower in the settled + filtered wine, while all other wines had similar concentrations. Fractional analysis revealed that the main differences occurred in the high-molecular-weight fraction (>180 kDa).

These differences can be directly linked to the applied treatments. On the one hand, must settling promotes the sedimentation of suspended solid particles, which may act as an important source or reservoir of polysaccharide-rich material. On the other hand, filtration can selectively retain high-molecular-weight polysaccharides that are unable to pass through the filter matrix, thereby reducing their presence in the final wine [33]. Overall, the combination of both processes appears to have a cumulative effect, leading to a more pronounced reduction in total polysaccharide content in the settled + filtered wines.

### 3.4.  Colloidal Properties

Wine is a complex matrix containing insoluble particles in suspension that can form colloids. These colloids, which range in size from the micrometre to the nanometre scale, can be divided into two categories. Associated colloids (or micelles) arise from van der Waals forces or other low-energy interactions between non-polar regions of molecules (e.g., phenolic compounds, colouring matter, cellulose microfibres, bacterial and yeast components, ferric phosphate, and clarifying agents). Macromolecular colloids, on the other hand, result from the aggregation of protein and/or polysaccharide molecules present in the medium [11].

Excessive colloids may form visible aggregates that can cause turbidity in wine [30]. Novel techniques have recently been applied to determine the colloidal properties of red [22,34] and white wines [35]. In contrast, few studies have examined the colloidal composition of sparkling wines [7,36].

The Nanoparticle Tracking Assay (NTA) was used to determine the concentration and particle size percentiles (Table 2). No differences in modal particle size were observed among the groups, whereas differences were detected in particle concentrations. Specifically, non-settled wines had higher colloid concentrations, which may increase turbidity risk and alter wine flavour [30].

The settled + non-filtered wine showed a lower particle concentration than the settled + filtered wine, probably because the insoluble particles absorb colloid-forming molecules prior to their precipitation [7].

The percentiles shown in Table 2 represent the diameters of the molecules in the sample. In all cases, the settled + filtered wine had larger diameters. Percentiles D10, D50 and D90 indicate the percentage of colloids found as a function of the size range. For percentile D90, non-settled wines showed smaller colloidal diameters than settled wines. A similar trend was observed for percentile D50 whereas no clear pattern was detected for percentile D10. Two-way ANOVA revealed a significant interaction between treatments (*p* < 0.05), suggesting that colloidal particle size may be influenced by the combined effect of both treatments.

### 3.5.  Foaming Properties

**Figure 4:**
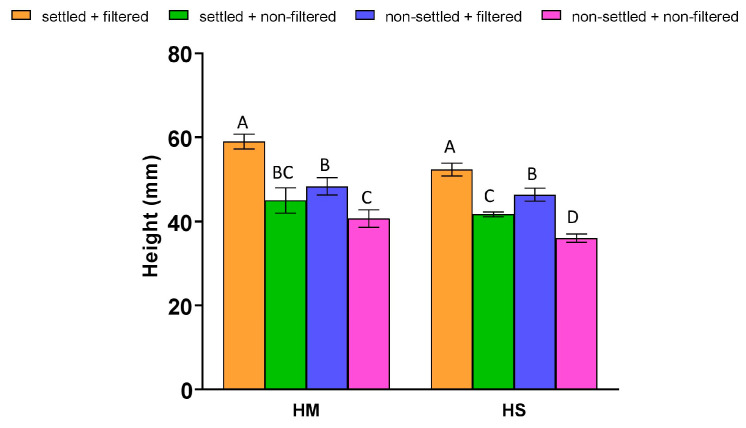
Foam properties. Results are expressed as mean ± SD of three replicates. HM: maximum height of the foam; HS: stable height of the foam. Different letters indicate the existence of a statistical difference (*p* < 0.05).

Figure 4 presents the Mosalux results. Maximum foam height (HM) and stable foam height (HS) were higher in the settled + filtered sparkling wines, while the non-settled + non-filtered wines exhibited lower levels for both parameters. These findings, together with the colloidal data, suggest that must settling and pre-bottling filtration may promote the elimination of large colloids that act as foam antagonists.

A two-way ANOVA was performed to evaluate potential interactions between both treatments affecting foaming parameters. A significant interaction between filtration and settling was observed for the HM parameter, whereas for HS the effects were independent, as no significant interaction was detected.

### 3.6.  Composition of Volatile Compounds

**Figure 5:**
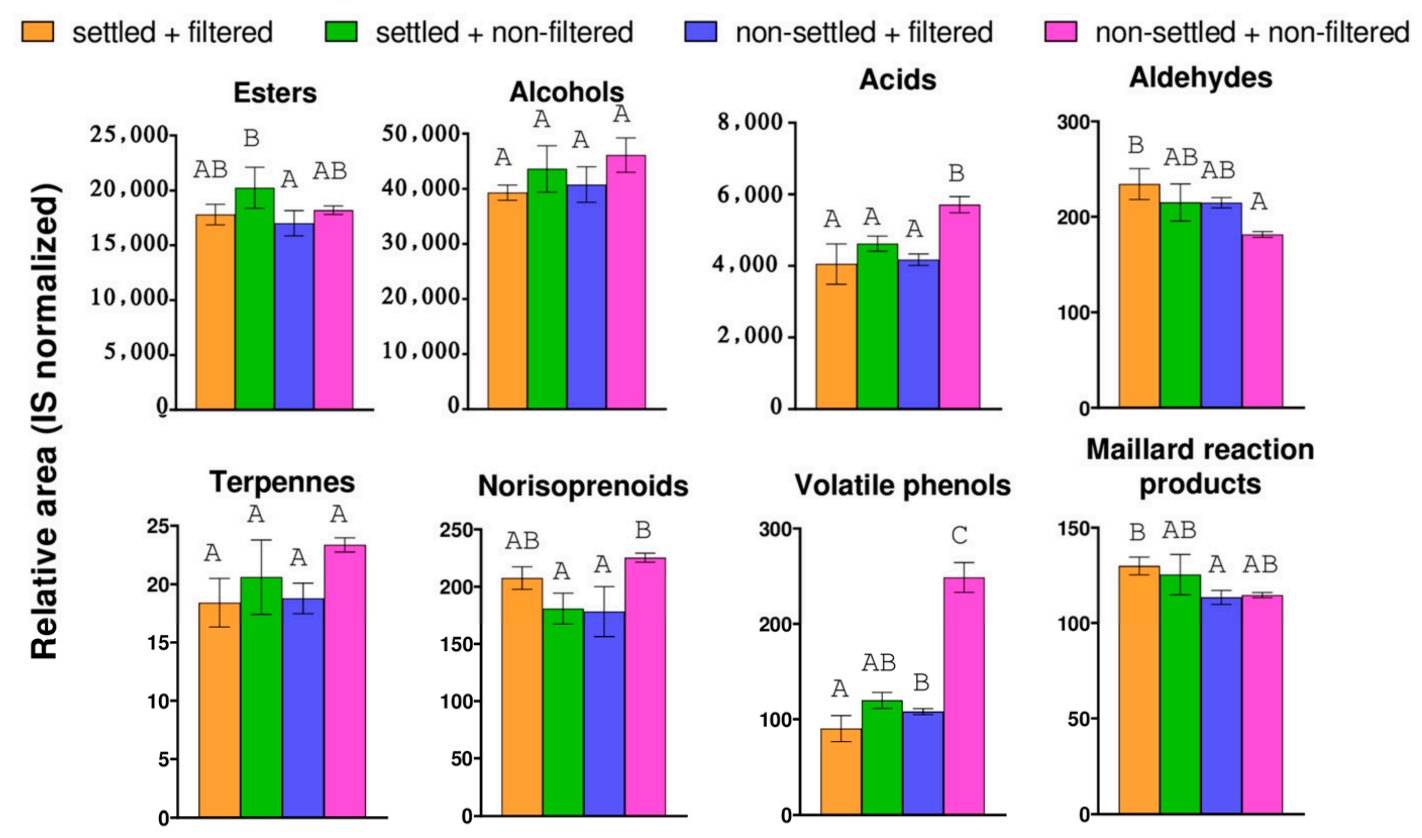
Volatile compound families. Results for each volatile compound family are expressed as the mean ± SD of three replicates. The value of a specific family corresponds to the sum of the GC–MS peak areas of all compounds classified within that family. These summed areas were then normalized to the peak area of the internal standard (4-methyl-2-pentanol). Different letters indicate statistically significant differences (*p* < 0.05).

A total of 62 compounds were quantified; of these, 57 were positively identified, and the remaining five were tentatively identified (isopentyl methoxy acetate, butyl ethyl succinate, diethyl malate, undecanol, and 2,5-dimethylbenzaldehyde). To better understand the results, the molecules were divided into eight chemical families: esters, alcohols, acids, aldehydes, terpenes, norisoprenoids, volatile phenols and Maillard reaction products. The total concentrations of volatile compounds found for each experimental condition are shown in Figure 5, while individual compounds within each family are reported in Supplementary Table S2. No statistically significant differences were observed between treatments in the alcohol or terpene families.

**Figure 6:**
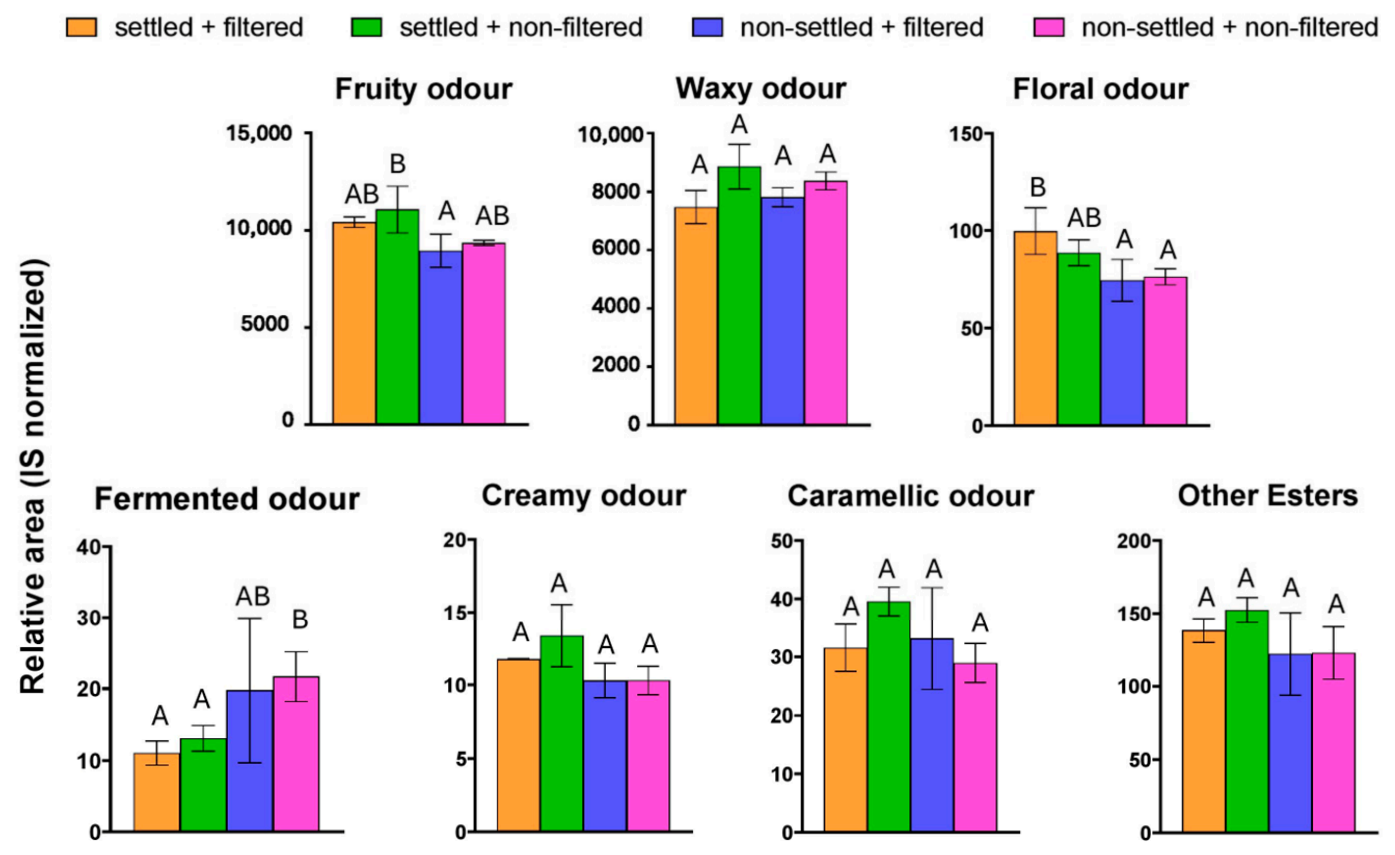
Volatile ester subfamilies. Results for each volatile ester subfamily are expressed as the mean ± SD of three replicates. The value of a specific subfamily corresponds to the sum of the GC–MS peak areas of all compounds classified within that family. These summed areas were then normalized to the peak area of the internal standard (4-methyl-2-pentanol). Different letters indicate statistically significant differences (*p* < 0.05).

The esters group comprised the largest number of chemical molecules. The esters were divided into seven subfamilies according to odour description based on the Good Scents Company database (https://www.thegoodscentscompany.com/, accessed on 25 January 2026). Supplementary Table S2 lists the compounds included in each of these subfamilies. The individual concentrations of all the analysed volatile compounds are shown in Supplementary Table S3. As Figure 6 shows, differences were observed in the fruity, floral and fermented ester families. Wines produced from settled must exhibited a greater presence of fruity and floral ester subfamilies. As previously reported [37], grape solid particles contain esterases responsible for the degradation of esters. Since settling removes a substantial proportion of suspended solid particles from the must, esterase activity in clarified musts is expected to be lower. This reduced hydrolytic capacity may explain the higher fruity and floral ester quantity observed in settled musts.

The family of volatile acids includes short-, medium-, and long-chain fatty acids. Elevated amounts of these compounds are consistently associated with undesirable sensory attributes. When present above their perception thresholds, they are commonly linked to rancid, sweaty or otherwise unpleasant aromas, which negatively impact the product’s overall aromatic quality [38]. In the present study, the non-settled + non-filtered sparkling wine presented significantly higher abundance of these compounds, a consistent result with previous reports indicating that fatty acid levels may increase when settling is not performed prior to fermentation [39].

Aldehyde amounts were higher in the settled + filtered sparkling wine. This family of aromatic compounds mainly comprises benzaldehyde and 2,5-dimethyl-benzaldehyde, which are described in the literature as pleasant bitter almond or Amarena cherry nuance descriptors [40,41]. In sparkling wines, their presence has been reported to increase during ageing through Maillard reaction products [42]. Previous studies [40,43] indicate that higher benzaldehyde levels can occur through two main pathways: enzymatic and/or chemical aldehyde oxidation. In settled + filtered wines, both the enzymatic and chemical pathways are more effectively controlled. Settling facilitates the early removal of enzyme-rich suspended solids, thereby reducing the potential for enzyme-mediated reactions. Moreover, SO_2_ addition at this stage inhibits oxidative enzymes and limits chemical oxidation, thereby enhancing overall wine stability.

Norisoprenoid amounts were higher in the non-settled + non-filtered sparkling wines than in the groups subjected to settling or filtering alone. Interestingly, norisoprenoids in the settled + filtered sparkling wines showed intermediate values, with no statistically significant differences relative to the other experimental groups. Previous studies have reported that norisoprenoid concentrations increase during sparkling wine ageing [44]. These compounds are formed through the enzymatic degradation of carotenoids [45]. As hypothesized above, non-settled + non-filtered wines may exhibit greater enzymatic activity due to the enhanced extraction of pulp-derived components [46], which may potentially explain the higher norisoprenoid levels observed in this group.

The quantities of volatile phenols showed the greatest significant differences between the non-settled + non-filtered wines and the others. Three volatile ethyl-phenols (phenol, 4-ethylguaiacol and 4-ethyl-phenol), commonly associated with wine faults, were detected in the samples. Ethyl-phenols originate from the enzymatic decarboxylation of hydroxycinnamic acids performed by microorganisms such as *Brettanomyces bruxellensis* [47,48]. Solid particles present in the non-settled wine may be responsible for the greater hydroxycinnamic acid solubilisation which, combined with a higher level of uncontrolled lees from the non-filtration and low sulphurous addition, may have helped the development of spoilage microorganisms such as *B. bruxellensis* [49].

Maillard reaction products are a heterogenous family of pleasant compounds associated with roasted, toasted, caramel and nutty aromas [50]. In sparkling wines, their presence is linked to on-lees ageing. In this study, three compounds were identified (furfural, 5-methylfurfural and ethyl-2-furoate), as well as benzaldehyde, which was classified as an aldehyde. The formation of Maillard reaction products depends on the sugar concentration and/or amino compounds [51]. As Table 1 shows, residual sugar levels were higher in the settled wines, which may have favoured a more rapid formation of Maillard reaction products and could explain the small but statistically significant differences shown in Figure 4.

### 3.7.  Volatile Compounds/Principal Component Analysis

**Figure 7:**
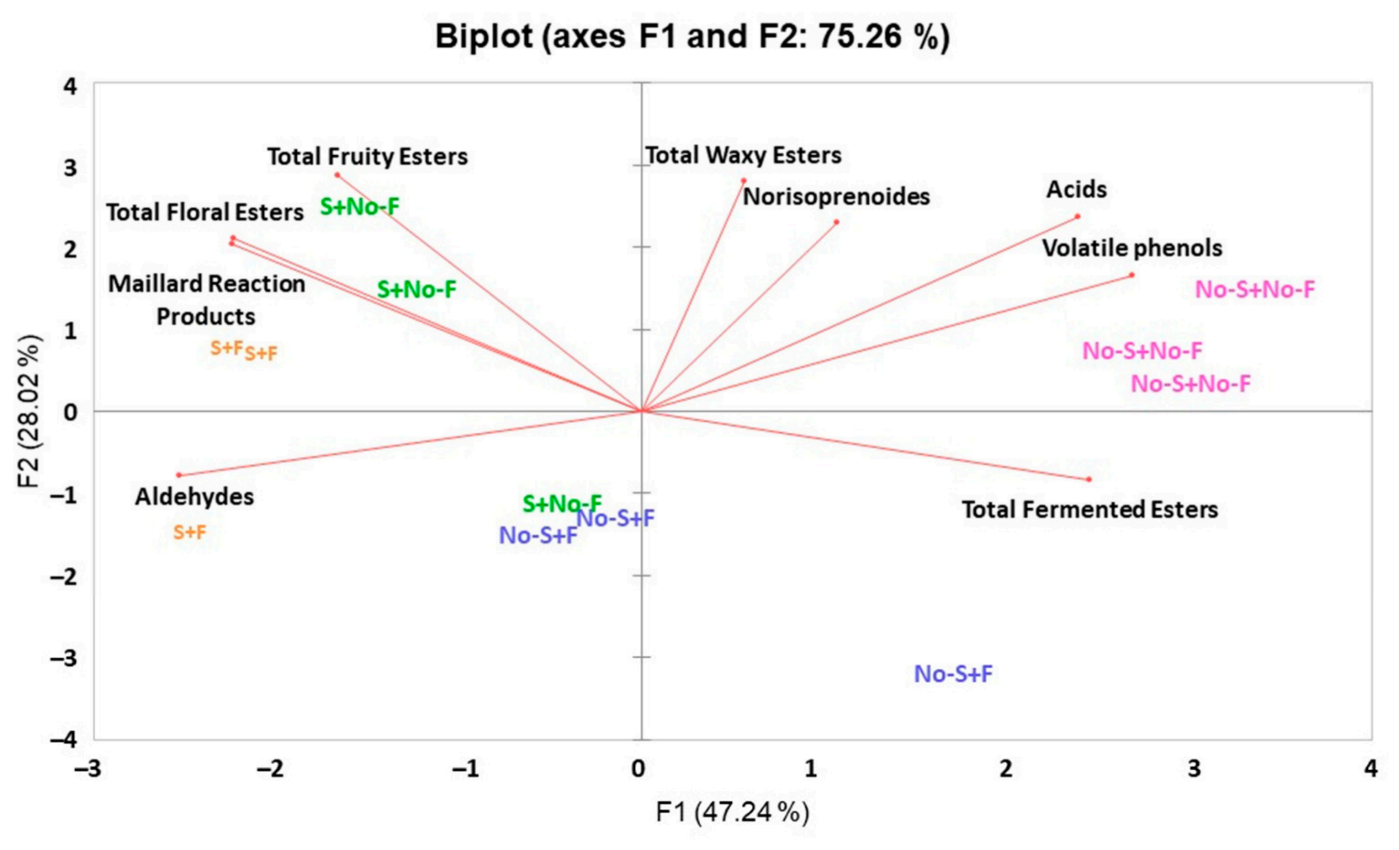
Plot of varimax-rotated principal component analysis for the different sparkling wines. Red vectors with black labels: loaded variables. S + F: settled + filtered sparkling wines. S + No-F: settled + non-filtered sparkling wines. No-S + F: non-settled + filtered sparkling wines. No-S + No-F: non-settled + non-filtered sparkling wines (*p* < 0.05).

To better understand the differences among the four experimental conditions, we performed principal component analysis. Figure 7 shows the varimax-rotated plot for this analysis. The first principal component (PC1) explained 47.24% of the variance, while the second component (PC2) explained 28.02%, resulting in an aggregate variance of 75.26% for the two components. The loadings are represented as arrows, whose length and direction indicate the contribution made by both components. PC1 showed a significant correlation with all variables except total fermented esters and aldehydes. Specifically, it was positively correlated with Maillard products, total floral esters, total fruity esters, total waxy esters, norisoprenoids, acids and volatile phenols. In turn, PC2 showed a significant positive correlation with total waxy esters, norisoprenoids, acids, volatile phenols and total fermented esters and a negative correlation with aldehydes, Maillard products, total floral esters and total fruity esters.

Non-settled + non-filtered sparkling wines are clearly isolated from all other sparkling wines and positively correlated with volatile phenols, acids and total fermented esters, which are broadly associated with unpleasant aromas. In contrast, settled + filtered sparkling wines, located in the opposite region of the PCA, have strong positive correlations with aldehydes and Maillard products, which are typically associated with pleasant aromas.

In the graphical representation, the settled + non-filtered and non-settled + filtered sparkling wines occupy intermediate positions and are interspersed. Both groups are closer to the settled + filtered wines than to the non-settled + non-filtered wines. While the non-settled + filtered group shows no positive correlation with any loaded variable, the settled + non-filtered wines display a slight positive association with total fruity esters.

### 3.8.  Sensory Assessment

Table 3 presents the results of the sensory triangular tests, including the *p*-values calculated from the proportion of correct responses. A *p*-value below 0.05 was considered indicative of a statistically significant difference for a given test. The results show that the non-settled + non-filtered wine was distinguishable from both settled wines, but not from the other non-settled wine. The non-settled + filtered wine was distinguishable only from the settled + non-filtered wine. No significant differences were observed between the two settled wines.

**Figure 8:**
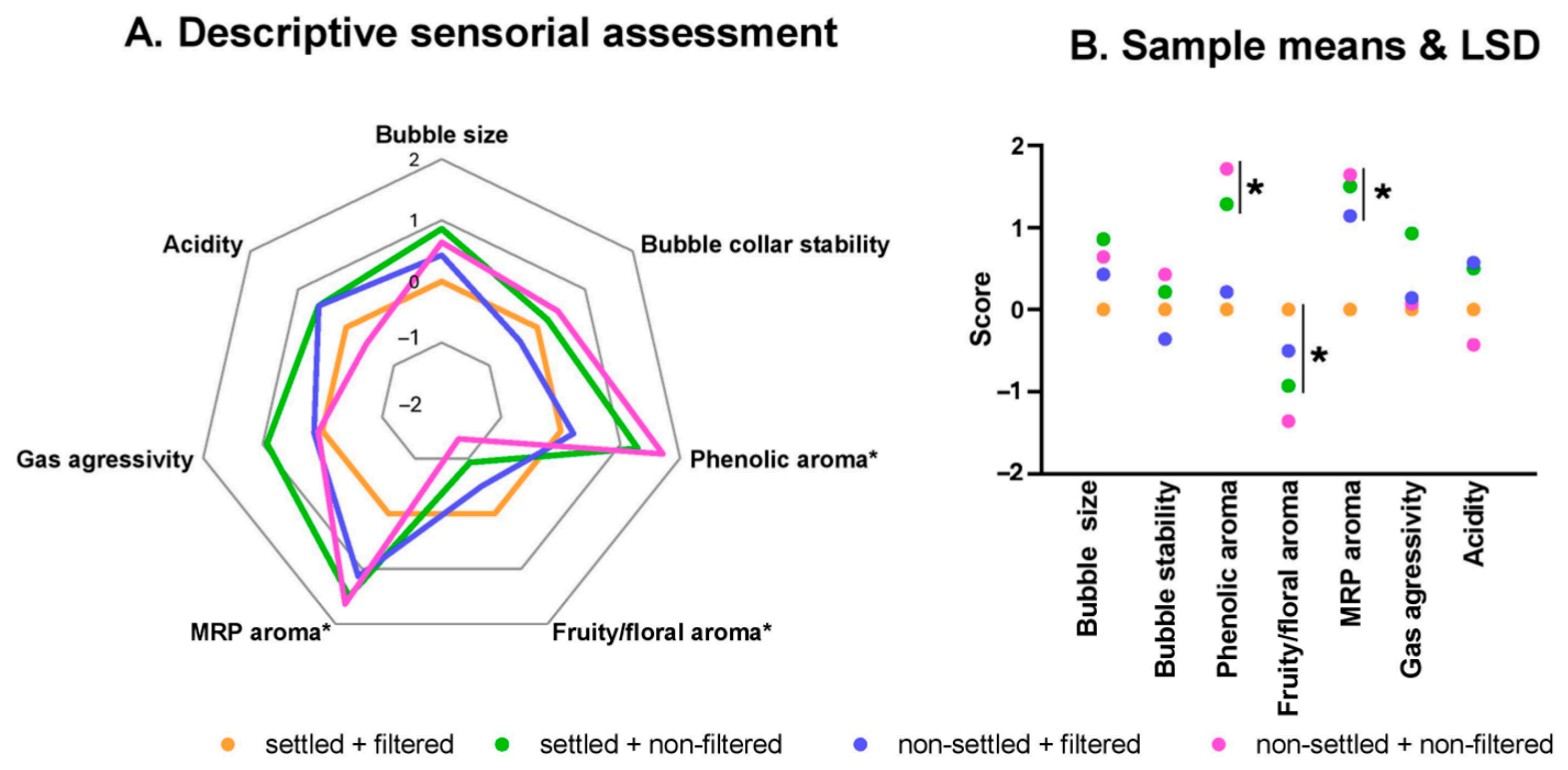
Descriptive sensorial assessment test. (**A**) shows the normalized results for the descriptive test. (**B**) shows the Sample means & LSD for every analyzed parameter. The asterisks (*) in (**A**) indicate the parameters for which significant differences were found (*p* < 0.05). Groups identified with an asterisk (*) in (**B**) show statistical differences with the ones outside the line (*p* < 0.05).

Figure 8A shows the normalised results from the descriptive test. Significant differences were observed for three parameters (MRP aroma, fruity/floral aroma, and phenolic aroma). Figure 8B shows the experimental groups between which the statistical differences were found.

A phenolic aroma that can also be described as an animal nuance [52] was stronger in the non-filtered wines, regardless of whether they had undergone settling prior to fermentation. These findings are broadly consistent with the results for GC-Headspace analysis.

Fruity/floral aromas were significantly lower in the non-settled + non-filtered sparkling wines. Although this result would be expected to relate to ester concentrations, only a similar pattern rather than a direct relationship was observed, probably because aroma perception is influenced by other aromatic compounds.

Finally, MRP aroma, or Maillard nuance, was lower in the settled + filtered wines. Pre-bottling filtration impacts the yeast population present inside the bottle, reducing the amount of yeast lees available for the autolysis process, which is directly related to the presence of these aromatic compounds. Interestingly, the volatile compounds assay found higher relative concentrations of MRP and aldehydes in the settled + filtered group, which contradicts the lower sensory perception. This apparent discrepancy suggests that concentrations of volatile compounds do not necessarily translate into a proportional sensory perception. Instead, it can be hypothesized that matrix composition and interactions among compounds can modulate aroma expression, as previously reported by several authors [3,53,54].

## 4. Conclusions

This study explored how two important oenological practices—must settling (using pectolytic enzymes) and pre-bottling filtration—influence sparkling wine production using the ancestral method. Both practices have recently been questioned by producers employing alternative winemaking approaches. The four wines obtained with and without these treatments showed substantial chemical differences only in total polyphenol index and titratable acidity (TTA). Protein concentrations after eighteen months were influenced by pre-bottling filtration, whereas no effect was attributed to must settling. The polysaccharide composition of the wines was highly affected when one or both treatments were applied.

Pre-bottling filtration, in combination with grape must settling, appeared to influence the colloidal composition of the resulting wines. Further studies are needed to better understand the implications of this phenomenon, particularly its potential effects on foam behaviour. In contrast, no clear differences were observed when settling was applied alone.

Both must settling and pre-bottling filtration improve the foamability of ancestral sparkling wines, since both techniques aid matrix stabilisation and proper foam development.

The composition of volatile compounds indicates that the volatile profile of ancestral sparkling wines is influenced by must settling prior to fermentation and, to a lesser extent, by filtration of the fermenting must before bottling. Must settling removes or prevents the formation of volatile compounds responsible for some of the most undesirable aromas. These findings suggest that certain oenological practices, although increasingly popular among some winemakers, can significantly alter the flavour of the wines and consequently the consumer’s perception.

Sensory assessment showed that applying both techniques has a perceptible effect on the final wines. These techniques may therefore influence wine development during the ageing-on-lees process.

It should be noted that this study was conducted using a single grape cultivar, one vintage, and a specific settling protocol that included the addition of SO_2_ and enzymes. In addition, each winemaking treatment was performed as a single fermentation without independent biological replicates; due to the complexity of replicating the entire vinification process. For that reason, replications were conducted using independent bottles for analytical determinations. Although this approach allowed controlled treatment comparisons, the lack of fermentation replicates limits the statistical robustness of the results. Therefore, the findings should be considered specific to the settling protocol investigated, and future studies including biological replication of the complete vinification process and additional grape cultivars and vintages are needed to validate these results and improve our understanding of aroma development and foaming properties in sparkling wines produced by the ancestral method.

## Figures and Tables

**Table 1:** General parameters.

	Settled + Filtered				Settled + Non-Filtered				Non-Settled + Filtered				Non-Settled + Non-Filtered			
Ethanol Content (%)	10.9	±	0.2	A	11.2	±	0.1	A	10.9	±	0.1	A	10.9	±	0.1	A
pH	3.0	±	0.0	A	3.0	±	0.0	A	3.0	±	0.0	A	3.0	±	0.0	A
TPI	4.6	±	0.0	A	5.4	±	0.1	C	4.7	±	0.0	A	5.1	±	0.1	B
SO_2_ (mg/L)	19.3	±	0.6	B	19.3	±	0.6	B	2.3	±	1.2	A	3.3	±	0.6	A
Residual fermentable sugars (g/L)	0.5	±	0.1	C	0.4	±	0.0	B	0.2	±	0.0	A	0.2	±	0.0	A
Glycerol (g/L)	4.1	±	0.1	A	4.1	±	0.2	A	4.1	±	0.1	A	4.1	±	0.1	A
Titratable acidity (g of tartaric acid/L)	6.7	±	0.2	B	6.8	±	0.0	B	6.2	±	0.2	A	6.4	±	0.1	A
Tartaric acid (g/L)	5.6	±	0.1	C	5.4	±	0.1	BC	5.1	±	0.1	A	5.3	±	0.0	B
L-malic acid (g/L)	0.5	±	0.0	A	0.6	±	0.0	B	0.7	±	0.0	C	0.7	±	0.0	C
Citric acid (mg/L)	125.7	±	4.6	A	128.3	±	3.2	A	137.7	±	3.8	B	138.3	±	5.5	B
L-lactic acid (g/L)	N-D				N-D				N-D				N-D			
D-Lactic acid (g/L)	0.1	±	0.0	A	0.1	±	0.0	A	0.1	±	0.0	A	0.1	±	0.0	A
Gluconic acid (g/L)	0.1	±	0.0	A	0.1	±	0.0	A	0.1	±	0.0	A	0.1	±	0.0	A
Acetic acid (g/L)	0.1	±	0.0	A	0.1	±	0.0	A	0.1	±	0.0	A	0.1	±	0.0	A
Ammonium (g/L)	23.3	±	3.1	A	31.3	±	2.1	A	23.0	±	6.1	A	28.0	±	6.6	A
Primary amino nitrogen (mg/L)	17.3	±	1.5	A	43.0	±	3.5	C	30.7	±	3.2	B	44.7	±	4.5	C

Results are expressed as mean ± standard deviation of three replicates. N-D: Not determined. Different letters in a row indicate the existence of statistical differences (*p* < 0.05).

**Table 2:** Colloidal parameters.

		Settled + Filtered				Settled + Non-Filtered				Non-Settled + Filtered				Non-Settled + Non-Filtered				S × F
Colloidal concentration [million particles/mL]		410.6	±	63.7	B	251.7	±	37.2	A	599.1	±	62.0	C	575.5	±	53.1	C	ns
Colloidal diameter size (nm)	D10	131.6	±	6.0	C	113.3	±	3.0	A	128.5	±	3.1	BC	122.1	±	1.7	AB	*
	D50	276.9	±	9.4	C	211.5	±	6.8	B	209.9	±	11.2	AB	196.3	±	5.4	A	*
	D90	866.1	±	51.9	C	560.3	±	51.1	B	403.3	±	47.6	A	369.2	±	35.5	A	*

Results are expressed as mean ± standard deviation of three replicates. Different letters in a row indicate the existence of statistical differences (*p* < 0.05). S × F column indicates interaction between both treatments; * indicates a significant interaction between both treatments (*p* < 0.05); ns indicates no significant interaction for the studied parameter.

**Table 3:** Sensorial triangular tests.

*p* Value	Settled + Non-Filtered	Non-Settled + Filtered	Non-Settled + Non-Filtered
settled + filtered	0.088	0.79	0.0002
settled + non-filtered		0.03	0.0002
non-settled + filtered			0.79

Triangular sensorial assessment results. Significant *p* values (<0.05)indicate that for the given significant level, the panel was able to distinguish those two wines.

## Data Availability

The original contributions presented in this study are included in the article/supplementary material. Further inquiries can be directed to the corresponding author.

## Supplementary Materials

The following supporting information can be downloaded at https://www.mdpi.com/article/10.3390/foods15152661/s1: Table S1: CIE*L***a***b** coordinate parameters; Table S2: Identification, chemical family and subfamily of the 28 selected volatile compounds according to wine type and possible odour description according to the literature; Table S3: Relative concentrations of the 28 selected volatile compounds according to wine type.
