# Supplementary material for: Effects of Must Settling and Pre-Bottling Filtration in Ancestral Sparkling Wine Production
Source: Foods. 2026 Jul 29;15(15):2661. doi: 10.3390/foods15152661 (PMC13464383; doi:10.3390/foods15152661)
Supplement: Supplementary file 1 [file foods-15-02661-s001.zip › foods-4382024-supplementary.pdf]

Table S1. CIE*L*a*b** coordinates parameters.

	a*				b*				L*			
Settled + Filtered	-0.41	±	0.02	C	4.67	±	0.01	A	98.60	±	0.06	A
Settled + Non-Filtered	-0.90	±	0.07	A	5.52	±	0.06	C	98.60	±	0.10	A
Non-Settled + Filtered	-0.76	±	0.02	B	5.32	±	0.01	B	98.73	±	0.06	A
Non-Settled + Non-Filtered	-0.94	±	0.03	A	5.75	±	0.01	D	98.70	±	0.06	A

Results are expressed as mean ± standard deviation of three replicates. Different letters in a row indicate the existence of statistical differences ($p < 0.05$).

Table S2. Identification, chemical family and subfamily of the 28 selected volatile compounds according to wine type and possible odour description according to the literature.

LRI	Volatile compound	Chemical family	Chemical sub-family	Odour description
1025	2-methyl-ethyl butyrate	Esters	Fruity	green apple, tropical fruit
1038	Ethyl isovalerate	Esters	Fruity	sweet, fruity, spice, metallic
1096	Isoamyl acetate	Esters	Fruity	Banana
1222	Ethyl hexanoate	Esters	Fruity	Sweet, banana, waxy
1287	Ethyl (E)-3-hexenoate	Esters	Fruity	Passion fruit, pineapple, rum and brandy, honey
1323	Ethyl heptanoate	Esters	Fruity	Pineapple, cognac
1332	Ethyl lactate	Esters	Fruity	Tart, buttery, butterscotch
1378	Methyl octanoate	Esters	Waxy	Vegetable, herbal
1428	Ethyl octanoate	Esters	Waxy	Musty, soapy, cognac
1475	Ethyl 7 octenoate	Esters	Other	N/D
1536	Ethyl 2-hydroxy-4-methylvalerate	Esters	Fruity	Fresh blackberry
1564	Isopentyl methoxyacetate*	Esters	Other	N/D
1584	Methyl decanoate	Esters	Fermented	Fermented
1629	Ethyl decanoate	Esters	Waxy	Oily
1639	Butyrolactone	Esters	Creamy	Milky, peach-like
1652	Isoamyl octanoate	Esters	Fruity	Pineapple, coconut
1669	Diethyl succinate	Esters	Fruity	Tropical and passion fruit
1682	Ethyl trans-4-decenoate	Esters	Fruity	Pineapple, pear
1772	Diethyl glutarate	Esters	Other	N/D
1781	Ethyl phenylacetate	Esters	Floral	Liquorice, cocoa, balsamic
1785	Butyl ethyl succinate*	Esters	Other	N/D

1813	2-phenyl ethyl acetate	Esters	Floral	Liquorice, cocoa, balsamic
1832	Ethyl dodecanoate	Esters	Waxy	Waxy, oily
1855	Isopentyl decanoate	Esters	Waxy	Waxy, dirty green, banana
2037	Ethyl tetradecanoate	Esters	Waxy	Sweet, waxy
2044	Diethyl malate*	Esters	Caramellic	Caramellic, brown sugar
2105	Ethyl 3 hydroxytridecanoate	Esters	Fruity	Citrus, grape, cranberry
2241	Ethyl hexadecanoate	Esters	Waxy	Buttery, vanilla, balsamic
2250	γ-Carboethoxy-γ-butyrolactone	Esters	Other	
2408	Ethyl hydrogen succinate	Esters	Other	N/D
1071	Isobutanol	Alcohols	-	Fusel, whisky
1204	Isoamyl alcohol	Alcohols	-	Fusel, fermented, fruity
1352	Hexanol	Alcohols	-	Green, fruity, apple
1459	Heptanol	Alcohols	-	Solvent, oily, green
1493	2-ethyl-1-Hexanol	Alcohols	-	Citrus, oily, sweet
1768	Undecanol*	Alcohols	-	Soapy, fresh, laundered cloth
1929	Phenylethyl Alcohol	Alcohols	-	Rose
1443	Acetic acid	Acids	-	Vinegar, sour
1844	Hexanoic acid	Acids	-	Cheesy, sour, fatty
1959	2-ethyl-Hexanoic acid	Acids	-	N/D
2060	Octanoic acid	Acids	-	Rancid oil, cheesy, fatty
2281	Decanoic acid	Acids	-	Rancid, fatty, sour
2344	9-Decenoic acid	Acids	-	Waxy, green, fatty, soapy
1521	Benzaldehyde	Aldehydes	-	Almond, cherry, nutty
1619	3-methyl-benzaldehyde	Aldehydes	-	Almond, cherry, bitter
1712	4-ethyl-Benzaldehyde	Aldehydes	-	Almond, cherry, bitter
1822	2,5-dimethyl-benzaldehyde*	Aldehydes	-	N/D
1467	Nerol oxide	Terpenes	-	Green, vegetative, floral
1538	Linalool	Terpenes	-	Citrus, floral, orange
1704	α-Terpineol	Terpnes	-	Pine, resinous, citrus
1529	Vitispyrane a	Norisoprenoids	-	N/D
1532	Vitispyrane b	Norisoprenoids	-	N/D
1654	Acetophenone	Norisoprenoids	-	Sweet, nutty, cherry
1824	β-Damascenone	Norisoprenoids	-	Woody, sweet, berries
1837	3,4-Dimethylacetophenone	Norisoprenoids	-	N/D
1746	TDN	Norisoprenoids	-	Liquorice
2008	Phenol	Volatile phenols	-	Plastic, rubbery
2032	4-Ethylguaiacol	Volatile phenols	-	Spicy, clove, medicinal
2180	4-ethyl-Phenol	Volatile phenols	-	Smoke, animal
1453	Furfural	Maillard reaction products	-	Woody, bready, caramellic
1570	5-methylfurfural	Maillard reaction	-	Coffee, bready, caramellic

https://doi.org/10.3390/foods15152661

		products		
1611	Ethyl 2 furoate	Maillard reaction products	-	Burnt, balsamic, fruity

Note: LRI: linear retention index. Compounds marked with an asterisk (*) have been tentatively identified through spectral coincidence with NIST Library (Gaithersburg, MD, USA). Odour description, and sub-family assigned according to the database the good scents company (http://www.thegoodscentscompany.com/).

Table S3. Relative concentrations of the 28 selected volatile compounds according to wine type.

Volatile compound	Settled + Filtered	Settled + non-filtered	Non- settled + Filtered	Non-settled + non-filtered
2-methyl-ethyl butyrate	44.62 ± 8.10 A	39.94 ± 5.64 A	32.26 ± 3.72 A	31.86 ± 1.02 A
Ethyl isovalerate	101.19 ± 16.54 A	90.34 ± 13.26 A	75.49 ± 6.10 A	76.99 ± 6.12 A
Isoamyl acetate	474.61 ± 106.00 B	452.22 ± 73.20 B	320.86 ± 16.27 A	340.27 ± 27.68 A
Ethyl hexanoate	5934.43 ± 66.51 A	6518.60 ± 870.22 A	5486.35 ± 469.47 A	5623.61 ± 58.06 A
Ethyl (E)-3-hexenoate	19.83 ± 3.56 A	19.77 ± 1.93 A	16.17 ± 2.16 A	17.24 ± 1.27 A
Ethyl heptanoate	19.89 ± 3.25 A	19.86 ± 2.40 A	19.77 ± 1.99 A	22.70 ± 0.65 A
Ethyl lactate	203.24 ± 40.42 A	187.07 ± 23.37 A	172.24 ± 11.59 A	184.88 ± 15.07 A
Methyl octanoate	44.38 ± 5.76 A	52.07 ± 3.78 A	54.45 ± 6.11 A	67.67 ± 3.92 B
Ethyl octanoate	6321.65 ± 393.53 AB	7438.40 ± 712.46 B	6141.68 ± 524.68 A	6674.30 ± 132.25 AB
Ethyl 7 octenoate	11.68 ± 0.88 B	11.51 ± 0.99 B	3.72 ± 0.60 A	4.29 ± 0.28 A
Ethyl 2-hydroxy-4-methylvalerate	31.74 ± 5.57 A	31.14 ± 2.78 A	34.91 ± 4.07 A	41.06 ± 2.67 A

Isopentyl methoxyacetate*	6.81 ± 1.11	A	7.26 ± 0.74	A	6.28 ± 0.26	A	7.41 ± 0.70	A
Methyl decanoate	11.06 ± 1.68	A	13.13 ± 1.79	A	19.78 ± 10.12	AB	21.70 ± 3.48	B
Ethyl decanoate	1011.06 ± 180.69	A	1269.36 ± 166.35	A	1471.06 ± 248.06	A	1481.61 ± 171.76	A
Butyrolactone	11.77 ± 0.05	A	13.40 ± 2.14	A	10.32 ± 1.18	A	10.32 ± 0.96	A
Isoamyl octanoate	28.05 ± 4.13	A	32.92 ± 3.75	A	36.76 ± 6.83	A	36.48 ± 4.36	A
Diethyl succinate	2956.39 ± 106.44	AB	3060.80 ± 236.95	B	2345.13 ± 359.88	A	2620.69 ± 46.40	AB
Ethyl trans-4-decenoate	593.65 ± 13.42	B	604.09 ± 71.35	B	390.36 ± 1.96	A	346.74 ± 24.54	A
Diethyl glutarate	2.63 ± 0.39	A	2.60 ± 0.13	A	3.08 ± 0.33	A	3.65 ± 0.06	A
Ethyl phenylacetate	43.78 ± 3.83	A	41.53 ± 3.53	A	35.54 ± 4.41	A	39.41 ± 2.38	A
Butyl ethyl succinate*	4.74 ± 0.61	A	5.05 ± 0.29	A	4.15 ± 0.62	A	5.21 ± 0.46	A
2-phenyl ethyl acetate	56.00 ± 8.24	B	47.10 ± 3.12	AB	39.00 ± 6.46	A	36.92 ± 1.77	A
Ethyl dodecanoate	24.00 ± 6.74	A	27.61 ± 5.45	AB	37.12 ± 5.37	B	40.91 ± 4.88	B
Isopentyl decanoate	15.06 ± 4.16	A	17.81 ± 6.89	A	19.04 ± 3.82	A	20.34 ± 2.46	A
Ethyl tetradecanoate	28.01 ± 10.26	A	28.27 ± 9.14	A	42.45 ± 6.82	A	40.89 ± 3.98	A
Diethyl malate*	31.51 ± 4.05	A	39.51 ± 2.47	A	33.13 ± 8.76	A	28.92 ± 3.36	A
Ethyl 3 hydroxytridecanoate	11.86 ± 2.82	A	13.07 ± 2.13	A	13.98 ± 2.81	A	13.51 ± 1.05	A

Ethyl hexadecanoate	23.18	±	3.81	A	24.39	±	9.42	A	40.22	±	5.38	B	38.46	±	3.35	B
γ-Carboethoxy-γ-butyrolactone	21.71	±	2.25	A	24.95	±	0.80	A	21.64	±	4.33	A	18.50	±	2.28	A
Ethyl hydrogen succinate	90.63	±	3.93	A	101.02	±	6.76	A	83.29	±	25.84	A	83.74	±	15.90	A
Isobutanol	191.28	±	12.61	A	208.02	±	1.68	AB	212.74	±	24.09	AB	246.43	±	18.27	B
Isoamyl alcohol	33674.67	±	782.30	A	37642.53	±	3954.15	A	34899.09	±	2811.85	A	39336.10	±	3172.60	A
Hexanol	437.46	±	2.89	A	461.02	±	16.77	A	531.64	±	75.47	AB	614.47	±	3.83	B
Heptanol	96.54	±	0.10	A	106.89	±	16.13	A	107.49	±	10.97	A	118.38	±	9.17	A
2-ethyl-1-Hexanol	75.34	±	5.97	AB	62.95	±	7.09	A	76.62	±	13.06	AB	90.75	±	7.40	B
Undecanol*	13.10	±	1.75	A	13.31	±	1.07	A	14.21	±	2.38	A	19.43	±	0.15	B
Phenylethyl Alcohol	4825.64	±	581.39	A	5118.64	±	295.06	A	4933.05	±	473.82	A	5708.03	±	165.05	A
Acetic acid	143.22	±	3.25	A	157.05	±	22.52	A	135.27	±	14.27	A	150.73	±	11.78	A
Hexanoic acid	882.05	±	127.77	A	965.54	±	73.73	A	920.62	±	95.21	A	1156.65	±	31.78	B
2-ethyl-Hexanoic acid	3.33	±	0.38	A	2.71	±	0.50	A	2.30	±	0.35	A	2.75	±	0.35	A
Octanoic acid	2431.77	±	341.31	A	2705.31	±	132.37	A	2347.47	±	28.29	A	3178.40	±	150.60	B
Decanoic acid	469.84	±	107.08	A	617.69	±	67.80	A	584.82	±	158.10	A	1086.52	±	69.03	B
9-Decenoic acid	123.00	±	11.18	A	173.26	±	19.39	B	185.26	±	8.28	B	135.88	±	8.60	A

Benzaldehyde	17.65	±	1.64	A	28.99	±	2.50	B	23.08	±	3.52	AB	24.56	±	0.35	B
3-methyl-benzaldehyde	0.49	±	0.06	A	0.51	±	0.08	A	0.35	±	0.09	A	0.42	±	0.13	A
4-ethyl-Benzaldehyde	4.28	±	1.62	A	4.53	±	1.25	A	2.29	±	0.72	A	2.61	±	0.16	A
2,5-dimethyl-benzaldehyde *	211.89	±	18.38	B	181.06	±	17.97	AB	189.10	±	2.62	B	153.99	±	2.48	A
Nerol oxide	5.73	±	0.69	A	5.31	±	1.28	A	4.86	±	0.69	A	6.09	±	0.11	A
Linalool	6.22	±	0.54	A	7.75	±	1.39	A	7.55	±	0.26	A	8.92	±	0.50	A
α-Terpineol	6.46	±	0.92	A	7.54	±	0.82	AB	6.37	±	0.43	A	8.36	±	0.26	B
Vitispyrane a	102.66	±	5.57	B	85.19	±	5.57	A	83.71	±	7.99	A	95.59	±	3.59	A
Vitispyrane b	57.22	±	6.48	A	51.55	±	6.48	A	48.34	±	7.32	A	57.63	±	1.57	AB
Acetophenone	3.38	±	0.52	A	3.31	±	0.52	A	3.02	±	0.62	A	3.45	±	0.10	A
β-Damascenone	25.74	±	2.25	A	21.88	±	2.25	A	24.71	±	6.38	A	30.65	±	1.04	A
3,4-Dimethylacetophenone	3.43	±	1.29	A	3.65	±	1.29	A	1.49	±	0.62	A	1.86	±	0.13	A
TDN	15.09	±	1.65	A	15.21	±	1.65	A	16.88	±	2.71	A	36.11	±	2.41	B
Phenol	3.59	±	0.78	A	3.52	±	0.63	A	2.71	±	0.42	A	2.95	±	0.19	A
4-Ethylguaiacol	48.99	±	8.07	A	66.69	±	4.89	A	63.24	±	4.76	A	148.44	±	9.45	B
4-ethyl-Phenol	37.69	±	5.74	A	49.63	±	3.42	A	42.16	±	1.50	A	97.32	±	6.52	B

Furfural	71.79 ± 4.63 B	67.98 ± 6.63 B	61.35 ± 0.72 AB	54.63 ± 1.94 A
5-methylfurfural	2.00 ± 0.24 A	1.80 ± 0.13 A	1.82 ± 0.39 A	1.85 ± 0.03 A
Ethyl 2 furoate	56.17 ± 0.00 AB	55.63 ± 3.93 AB	50.30 ± 3.87 A	58.15 ± 1.15 B

Compounds marked with an asterisk (*) have been tentatively identified through spectral coincidence with NIST Library (Gaithersburg, MD, USA). Results are expressed as mean ± standard deviation of three replicates. Different letters in a row indicate the existence of statistical differences ($p < 0.05$).
